# Mineral Supplementation in Jade Perch (*Scortum barcoo*) Aquaponics with Lettuce: A Comparison with Hydroponics and RAS

**DOI:** 10.3390/ani15030317

**Published:** 2025-01-23

**Authors:** Maurício Gustavo Coelho Emerenciano, Joel Slinger, George Koster, Jarvis Aland, Paula Camargo Lima, Maja Arsic, Cathryn O’Sullivan

**Affiliations:** 1 The Commonwealth Scientific and Industrial Research Organisation (CSIRO), Agriculture & Food, Livestock & Aquaculture Program, Bribie Island Research Centre, Woorim 4507, Australia; joel.slinger@csiro.au (J.S.); george.koster@csiro.au (G.K.); jarvis.aland@csiro.au (J.A.); 2 The Commonwealth Scientific and Industrial Research Organisation (CSIRO), Agriculture & Food, Livestock & Aquaculture Program, Queensland Bioscience Precinct, St. Lucia 4067, Australia; paula.lima@csiro.au; 3 The Commonwealth Scientific and Industrial Research Organisation (CSIRO), Agriculture & Food, Sustainability Program, Queensland Bioscience Precinct, St. Lucia 4067, Australia; maja.arsic@csiro.au (M.A.);

**Keywords:** nutrients, circular economy, water quality, SPAD, freshwater aquaculture, land-based aquaculture

## Abstract

The global demand for local and high-quality food sources has increased in recent decades, driven by consumer preferences and a growing population. RAS, hydroponics and a ‘hybrid’ version, aquaponics, are food production techniques that could support such growing demand. The current study evaluated coupled aquaponics (Jade perch and lettuce) with and without mineral supplementation in comparison to standard cultivation methods, i.e., RAS and hydroponics. The mineral supplementation in aquaponics was carried out using a hydroponics commercial blend fertilizer (10% of the dosage utilised in the hydroponics treatment). Fish/plant performance, nutrient dynamics and water quality were evaluated for 9 weeks using twelve experimental units (~720 L in total; fish tank/reservoir + filtration systems/sump + floating raft-based plant tank). After two crops of plants, the results showcased the positive impact of mineral supplementation in aquaponics; however, the hydroponics treatment outperformed the two aquaponics treatments, e.g., with higher total plant wet weight and total plant length (*p* < 0.05). Fish in aquaponics with and without mineral supplementation presented similar growth performance as compared to RAS (*p* > 0.05). By comparing these different farming approaches, this study sought to provide insights into optimising aquaponics systems and advancing the integration of Jade perch into aquaculture practices.

## Simple Summary

Recirculating aquaculture systems (RAS) and hydroponics are indoor and controlled food production systems for aquatic proteins and vegetables. A ‘hybrid’ version is aquaponics, where these two techniques are combined, aiming to optimize footprint and nutrient utilization for a diversified production. The present study aimed to evaluate the performance, nutrient dynamics and water quality of a traditional (coupled) aquaponics setup using Jade perch (*Scortum barcoo*) and lettuce (*Lactuca sativa,* var. butterhead) with and without mineral supplementation in comparison to RAS (fish only) and hydroponics (plants only). In the current experimental conditions, fish in aquaponics had a similar performance compared to RAS. In terms of plant performance (two crops were evaluated), mineral supplementation in aquaponics helped to improve key metrics, e.g., total plant length and root length; however, hydroponics treatment presented a superior overall plant performance.

## 1. Introduction

The global demand for aquaculture products has surged dramatically in recent decades, driven by an increasing population and growing consumer preference for seafood [1]. In 2022, world aquaculture production reached a record high of 130.9 million tonnes, marking a 6.6 percent increase from 2020 [2]. This impressive growth reflects a broader trend, with aquatic animal production expected to rise by 10 percent by 2032 due to the expansion of aquaculture and the recovery of capture fisheries [2]. However, the intensification of traditional aquaculture systems to meet the ever-increasing demand has introduced a range of environmental challenges, including the excessive use of limited resources (i.e., water), water quality deterioration and loss of critical habitats (i.e., mangroves and wetlands) [3,4,5]. Therefore, there is an urgent need to explore more sustainable and integrated approaches to aquatic animal production as the industry continues to grow.

Aquaponics has emerged as a sustainable farming practice that integrates aquaculture and hydroponics into a mutually beneficial recirculating ecosystem [6,7,8]. In such systems, natural bacteria convert waste from farmed aquatic species—such as fish and crustaceans—into essential nutrients for plants [6]. The waste produced by the fish supplies most of the nutrients needed for plant growth, while the plants filter and purify the water, which is then recirculated back to the fish tank [6]. This symbiotic relationship not only reduces waste but also optimizes resource use, presenting a compelling alternative to conventional aquaculture intensification [7].

The benefits of aquaponics extend beyond the simultaneous production of plants and fish, improved water use efficiency and reduced aquaculture waste release. Another significant benefit is its reduced land footprint compared to traditional agriculture and inland aquaculture systems. Aquaponics can be implemented in areas with limited arable land, enabling the production of fresh food closer to consumers [9]. This proximity shortens supply chains, reduces transportation emissions and enhances food security [10].

Despite the advantages of conventional aquaponics, a key challenge is that plant species have varying nutrient and mineral requirements, which are often not in the ideal balance by the aquaculture wastes [11,12]. To address nutrient deficiencies and enhance plant growth performance in aquaponics systems, supplementation can be applied through foliar application [13,14,15], added directly to the culture water [16,17,18], or included in the fish diet [19,20,21], as demonstrated by several studies.

For example, Delaide et al. [16] found that, while the average fresh weight of lettuce shoots (*Lactuca sativa* ‘Sucrine’) exposed to hydroponic and aquaponic solutions was statistically similar, the treatment using 100% tilapia recirculating aquaculture system (RAS) water supplemented with high-purity mineral salts to match the nutrient concentrations of the hydroponic control showed a significant 39% increase in shoot mass. Similarly, Tsoumalakou et al. [22] demonstrated that an aquaponics system co-cultivating lettuce (*Lactuca sativa* var. Romana) and red tilapia (*Oreochromis* sp.) exhibited better performance with iron (Fe) and potassium (K) supplementation. The addition of both Fe and K sustained higher maximum values for all measured parameters compared to systems without supplementation. While Fe alone improved photosynthetic rates, capacity and the health of the photosynthetic apparatus, the addition of K was crucial for significantly enhancing lettuce yields compared to the no-supplementation control. Both fresh and dry biomass of aerial and root parts, as well as total leaf area, more than doubled with Fe and K supplementation compared to the control.

Recent interest in alternative freshwater fish species for aquaponics systems has highlighted the Jade perch, also called Barcoo grunter (*Scortum barcoo*), as a promising and potentially profitable candidate [23]. Jade perch is an Australian native species found in the Lake Eyre basin, from temperate (southern) through to tropical (northern) areas of inland New South Wales, South Australia, Queensland and Northern Territory. Its preferred habitat is the turbid waters of large freshwater rivers and waterholes [24]. It is an omnivorous species recognised for its robust health, rapid growth rates, high feed conversion efficiency, adaptability to diverse environmental conditions and excellent palatability [25,26,27]. These attributes make it an appealing choice for aquaponics integration, with the potential to enhance both the efficiency and sustainability of such systems.

The current land-based aquaculture industry for Jade perch in Australia is relatively small. In 2021/2022, the Australian Jade perch industry produced 74 tonnes of whole fish with a total value of $AUD 1.1 million [28]. However, Jade perch’s resilience and growth characteristics make it attractive as it can enable a more stable and productive aquaponics environment, benefiting both fish production and plant growth. From a broad perspective, this species is quite suitable for earthen pond production, and some Australian hatcheries export fingerlings to Asia (particularly China, Malaysia, Vietnam and Singapore) for growing out in land-based farms. It is generally believed that Jade perch aquaculture has the potential to grow significantly if given appropriate R&D and marketing support [29].

In this context, the present study aimed to evaluate the performance of both plants and fish in a traditional coupled aquaponics setup using jade perch and lettuce (*Lactuca sativa,* var. butterhead) with and without mineral supplementation. This study compared these results to those from a RAS featuring only Jade perch and a lettuce-based hydroponics system. Additionally, key nutrient dynamics and water quality were assessed. By comparing these different farming approaches, this study sought to provide insights into optimising aquaponics systems and advancing the integration of Jade perch into aquaculture practices.

## 2. Materials and Methods

### 2.1. Location and Experimental Design

The experiment was carried out at the Bribie Island Research Centre (BIRC/CSIRO, Bribie Island, QLD, Australia) in a translucent plastic-covered tunnel house, using natural photoperiod and no additional cover shade-cloth over the plant tanks. Based on data from the local weather monitoring station (managed by the Australian Bureau of Meteorology), the average incoming solar radiation over the duration of the experiment was 216.44 W m^−2^ day^−1^ (minimum and maximum of 39.4 and 295.1 W m^−2^ day^−1^, respectively) [30]. This is equivalent to a minimum and maximum of 8.55 and 47.3 μmol m^−2^ s^−1^, respectively.

The experiment assessed four treatments, i.e., four different food production techniques (with three replicates each): a coupled aquaponics system with no mineral supplementation (aquaponics; n = 3); a coupled aquaponics system with commercial mineral supplementation (aquaponics + mineral; n = 3); hydroponics (plant control; n = 3); and conventional RAS (fish control; n = 3). The experiment was run over nine weeks during March and April 2022, including 63 days of culture (DOC) for fish production in the RAS and aquaponics treatments and two crops of plants for the aquaponics and hydroponics treatments (24 and 27 DOC for crop 1 and 2, respectively).

**Figure 1:**
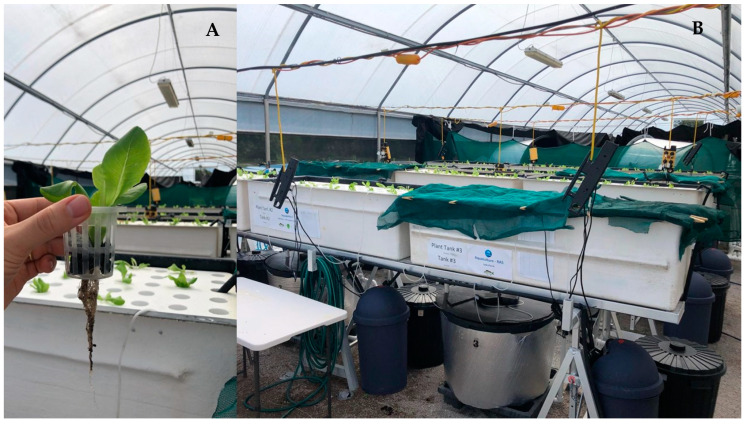
(**A**) Example of butter lettuce *Lactuca sativa* seedling utilised in this experiment; and (**B**) experimental system consisting of 12 individual units (three for RAS, three for hydroponics and six for aquaponics treatments.

Each experimental unit had a system volume of ~720 L and utilised the same infrastructure. The aquaponics and RAS treatment systems consisted of a fish tank (240 L useful volume), a rectangular fiberglass plant tank (380 L useful volume; this tank was retained with no plants present for the RAS treatment), mechanical filter (60 L clarifier tank) and biological filter/sump (40 L moving bed bioreactor containing ~0.02 m^3^ of C1 plastic media) (Figure 1). The hydroponics treatment utilised the fish tank, mechanical filter and biological filter (no media) recirculating reservoirs to provide a comparable water volume to the other treatments. In all treatments, the water was pumped (using a 45 W, 2200 L h^−1^ submersible pump) from the biological filter/sump to the plant tanks, back to the fish tanks and then onwards to the mechanical filter and biological filter/sump by gravity. A valve was used to regulate the flow through the system at a set point of 4 L min^−1^. At a flow rate of 4 L min^−1^, the water volume in the fish tanks was turned over once per hour (i.e., the full volume of water in the fish tanks was pumped out through the filters, the clarifiers, the plant tanks and returned to the fish tanks once every hour equating to a 2400% daily turnover). A 100-micron mesh was fitted on the clarifier outlets to retain particles before reaching the biological filter/sump.

Aeration was supplied to the fish tank (n = 2), plant tanks (n = 2) and the biofilter (n = 1) using rectangular air stones (~50 × 10 mm) connected to the BIRC radial blower main supply line. Prior to the commencement of the experiment, each biological filter was matured for 90 days using NH_4_Cl salt added daily. Aiming to keep temperatures within suitable culture conditions for Jade perch, two 300 W heaters were added to each system (~0.8 W/L). In RAS, sodium bicarbonate was routinely added, aiming to keep alkalinity > 100 mg L^−1^ and supporting the nitrification processes [26]. A similar process was used in aquaponics, but using a mixture of NaHCO_3_ (62.5%) and CaCO_3_ (37.5%). No alkalinity agents were added in hydroponics. Each system received water top-ups once or twice weekly (as needed) to account for plant evapotranspiration and minor system losses. The average water top-ups per experimental unit during the 63 days of the experimental period were: aquaponics (353.7 L), aquaponics with mineral supplementation (268.9 L), RAS (230.1 L) and hydroponics (152.9 L).

### 2.2. Fish

Jade perch fingerlings (approx. weight 2–5 g) were purchased from a local commercial hatchery and transported to BIRC facilities via oxygenated plastic bags with 5 ppt salinity water. Upon arrival, fingerlings were quarantined in a 17,000 L freshwater RAS at a stocking density of ~500 fish m^−3^ for 14 days. The RAS consisted of three 5000 L high-density polyethylene tanks, 50 µm mechanic filtration (automatic drum filter and sock filtration), fluidised bed biofilter, UV-sterilization and 1000 L sump. Water temperature was maintained via two commercial heat pumps (Sunlover, model Oasis X19, Hallam, VIC, Australia) using a ratio of ~1 W L^−1^. Optimal RAS water quality parameters were measured daily and maintained as followed: temp ~26 °C, dissolved oxygen >8 mg/L, salinity ~5 ppt, pH ~7.5, TAN < 0.5 ppm, NO_2_ < 0.5 ppm, NO_3_ < 80 mg/L and alkalinity > 100 mg L^−1^. During quarantine and rearing, fish were fed a commercial feed (45% protein 10% lipid, Ridley Aquafeeds, QLD) over a 12 h period via an automatic belt feeder (FIAP, Ursensollen, Germany).

For the stocking of the experimental systems, the fish were anesthetised using AQUI-S (Lower Hutt, New Zealand; 17 mg/L), weighed and transported to the experimental aquaponics area. All handling procedures were approved by the CSIRO Animal Ethics Committee (approved application number 2021-28).

Juvenile Jade perch (14.51 ± 0.92 g of initial weight) were randomly stocked into six individual aquaponics systems and three RAS. Both aquaponics systems (‘aquaponics’ and ‘aquaponics + mineral’) were coupled with the plants and fish raised in separate tanks. Fish tanks were stocked at a density of 32 fish per tank (134 juveniles m^−3^). Fish were fed three times a day using a commercial native fish diet (2 mm and 3 mm, 45% protein, 10% lipid, Ridley Aquafeeds, Narangba, QLD, Australia) at 5.5 to 2.4% body weight per day (high feeding rate at the beginning of the trial, decreasing over time). These levels met the ‘fish feed–plant’ nutrient ratio of approximately 2–3 g of fish feed/plant/day [31].

### 2.3. Plants

Fish culture in the aquaponics and RAS treatments began fourteen days prior to plant stocking in the aquaponics and hydroponics treatments to allow for nutrient accumulation in the aquaponics replicates. The plant seedlings (butter lettuce, *Lactuca sativa*) were sourced from a local nursery, with an initial aerial length of 11.56 ± 2.11 cm and 8.63 ± 1.53 cm for crop one and crop two, respectively. The seedlings were planted into the rectangular plant tank using a hydroponics floating raft system. Each plant tank (around 1 m height) was stocked at a density of ~18.5 plants/m^2^ (crop one, n = 20 and crop two, n = 17, to avoid excessive crowd effect in crop two). Each seedling was placed in a 60 × 55 mm clear net pot with small gravel (~20 mm) to hold the seedlings (Figure 1). For the hydroponics and aquaponics treatments, a commercial mix of hydroponic fertilizer was added to the replicates one day prior to the crop one plant stocking, following the manufacturer’s instructions (AutoPot, Braeside, VIC, Australia). According to calculations, manufacturer’s instructions and aiming to maintain an electrical conductivity (EC) of 1.2, each hydroponics replicate received 920 mL of AutoPot solution Part A and 920 mL of AutoPot solution Part B. The aquaponics treatment with mineral supplementation received 10% of the dose administered to the hydroponics treatment, meaning 92 mL of AutoPot solution Part A and 92 mL of AutoPot solution Part B. One day prior to the crop two stocking, 150 mL AutoPot solution Part A and Part B were added to the hydroponics treatments to return the EC levels to >1.2. A 10% dose of AutoPot solution Part A and Part B (i.e., 15 mL) was similarly added to the Aquaponics with mineral supplementation replicates.

### 2.4. Performance Parameters

The fish performance parameters recorded (for RAS and aquaponics treatments) were final individual weight (g), survival (%, number of fish at the start of the experiment/number of fish at the end of the experiment × 100), final biomass (kg tank^−1^, total weight of all fish in the tank at the end of the experiment (kg)), productivity (kg m^−3^, tank volume (m³)/final biomass (kg)), specific growth rate (SGR, %/day, [ln(final weight) – ln(initial weight)/days of culture] × 100) and apparent feed conversion rate (FCR, total feed given (kg)/total weight gain of fish (kg)). To assess growth and overall fish condition, biometrics (n = 5 fish per tank) were carried out on DOC 22, 37, 50 and 63 (final harvest). During the final harvest, fish was anesthetised and humanely killed using a lethal dose of AQUI-S (concentration 100 mg L^−1^).

For the plant performance, final wet weight (aerial + roots, g), total length (cm), root length (cm), aerial length (cm), number of leaves per plant (n) and length of the largest leaf (cm) were recorded. In addition, the leaf greenness index (SPAD) was analysed during harvest time in crop one and crop two using a chlorophyll meter (SPAD-502, Konica Minolta, Japan). Chlorosis (leaf yellowing due to loss of chlorophyll) can be indicative of a deficiency of a range of nutrients, and therefore, leaf greenness can be used as an indicator of some nutrient deficiencies. In this sense, SPAD readings were taken on older and younger leaves to provide an estimate of chlorosis due to a lack of mobile or immobile nutrients. For each treatment, a total of 15 young leaves and 15 older leaves were measured (5 plants per replicate tank). Three SPAD readings were taken on each leaf to give an average reading per leaf.

### 2.5. Water Quality Monitoring

Water quality parameters such as temperature (°C) and dissolved oxygen DO (mg L^−1^) were measured twice daily (8 am and 3 pm) with a handheld multiparameter (YSI ProDSS, YSI Incorporated, Yellow Springs, OH, USA). Electrical conductivity (EC) (µs cm^−1^), salinity (ppt), pH and turbidity (FNU) were measured daily (YSI ProDSS multiparameter, YSI Incorporated, Yellow Springs, OH, USA). Total ammonia nitrogen (TAN), nitrite (NO_2_), nitrate (NO_3_) and alkalinity were measured daily using colorimetry kits (API freshwater kit, API Fishcare, PA, USA) for husbandry intervention purposes only. Water samples (250 mL) were collected weekly for official data measurements of TAN, NO_2_, NO_3_, alkalinity and orthophosphate (PO_4_) using a photometer (YSI Photometer 9500, YSI Incorporated, Yellow Springs, OH, USA). Water samples (500 mL) were collected at four intervals throughout the trial (DOC 15, 37, 43 and 64) for K, Ca, Mg, Na and S measurements using the inductively coupled plasma mass spectrometry (ICP) method [32].

### 2.6. Statistical Analysis

Normality and homogeneity of variance were evaluated with the Kolmogorov–Smirnov and Levene tests, respectively [33]. Fish and plant performance (except SPAD) were analysed by one-way analysis of variance (ANOVA), followed by a Tukey post hoc test to detect significant differences among treatments at *p* < 0.05 [34]. SPAD data (i.e., old and young leaves) were analysed using two-way ANOVA with significant differences detected by Tukey post hoc test at *p* < 0.05 [34]. Descriptive statistics were calculated for the water quality parameters and presented as mean, standard deviation, maximum and minimum values. Percentage data was arcsine-transformed, but original values are presented.

## 3. Results

### 3.1. Water Quality Parameters

**Figure 2:**
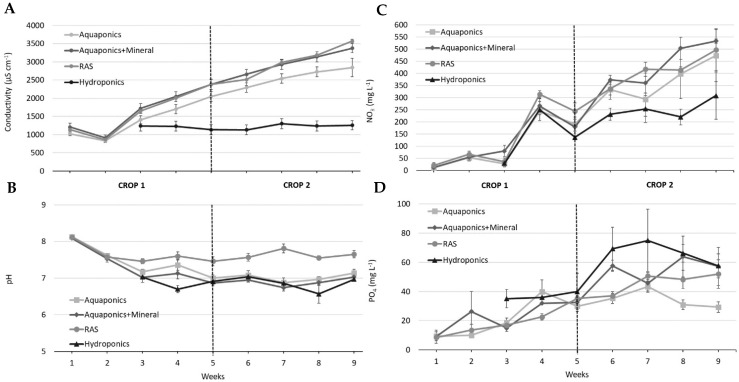
Fluctuations over time (mean values ± SE) of electrical conductivity (**A**), pH (**B**), nitrate (**C**) and orthophosphate (**D**) in Jade perch culture in aquaponics, aquaponics with mineral supplementation and RAS (fish control) during 63 days of experimental period, as well as hydroponics (plant control, 48 days of duration).

Water quality parameters and fluctuation over time are presented in Table 1 and Figure 2, respectively. Parameters such as temperature and DO were similar in all treatments and averaged ~26.1 °C and ~7.5 mg L^−1^. In terms of pH, lower values were observed in hydroponics (~6.8) compared to RAS (~7.5) and both aquaponics treatments (~7.2). As expected, electrical conductivity was impacted by mineral supplementation in aquaponics, with a slightly higher value compared to aquaponics without mineral supplementation (~2200 versus ~1900 µS cm^−1^, respectively); however, it was still comparable to RAS (~2200 µS cm^−1^). On the other hand, these values were higher when compared to hydroponics (~1200 µS cm^−1^), likely due to the addition of NaHCO_3_ and CaCO_3_ for alkalinity management. A similar trend occurred in turbidity, i.e., ~1.6 FNU in RAS and both aquaponics versus ~0.1 FNU observed in hydroponics.

Regarding the nitrogen compounds, the highest TAN values were observed in hydroponics, with an average of 2.9 mg L^−1^ (peak of 12 mg L^−1^ was detected) versus ~1.4 mg L^−1^ in RAS and both aquaponics. Nitrite averaged ~0.2 mg L^−1^ in both aquaponics and hydroponics and ~0.7 mg L^−1^ in RAS. The lowest nitrate concentration was found in hydroponics (average ~180 mg L^−1^), followed by aquaponics (~200 mg L^−1^) and RAS and aquaponics with mineral supplementation (~240 mg L^−1^). Alkalinity was higher in RAS, with an average of ~100 mg L^−1^, compared to ~50 mg L^−1^ in both aquaponics and hydroponics.

Regarding the essential plant nutrients, as expected, the mineral supplementation improved K, Ca, Mg and S levels in aquaponics. K was higher in hydroponics (~41 mg L^−1^), followed by aquaponics with mineral supplementation (~13 mg L^−1^), RAS (~10 mg L^−1^) and lastly aquaponics without mineral supplementation (~5 mg L^−1^). On the other hand, Ca was higher in aquaponics with mineral supplementation (~76 mg L^−1^) compared to aquaponics without mineral supplementation (~62 mg L^−1^), hydroponics (~45 mg L^−1^) and RAS (~18 mg L^−1^). Slightly higher levels of Mg were also found in aquaponics with mineral supplementation (average of ~13 mg L^−1^), followed by RAS (~12 mg L^−1^), aquaponics without mineral supplementation (~11 mg L^−1^) and hydroponics (~8 mg L^−1^). In terms of S, higher levels were found in aquaponics with mineral supplementation and hydroponics (average of ~1 mg L^−1^).

In terms of water quality fluctuations (Figure 2), electrical conductivity was stable in hydroponics and increased over time in both aquaponics treatments and RAS. pH trends over time were relatively similar. However, hydroponics treatment presented continuous levels of <6, compared to >7 in RAS and both aquaponics. Sodium concentrations also increased over time in the RAS, aquaponics and aquaponics + minerals treatments and were higher than in the hydroponics treatment. Although in different magnitudes, nitrate and orthophosphate increased over time in all treatments.

### 3.2. Fish and Plant Performance Parameters

The fish performance results are presented in Table 2. No statistical differences were detected in all the parameters analysed (*p* > 0.05). Final individual weight, SGR and final biomass averaged ~106 g, 3.15%/day and 3.3 kg tank^−1^. In addition, productivity, survival and FCR averaged 13.9 kg m^−3^, 100% and 1.23.

**Figure 3:**
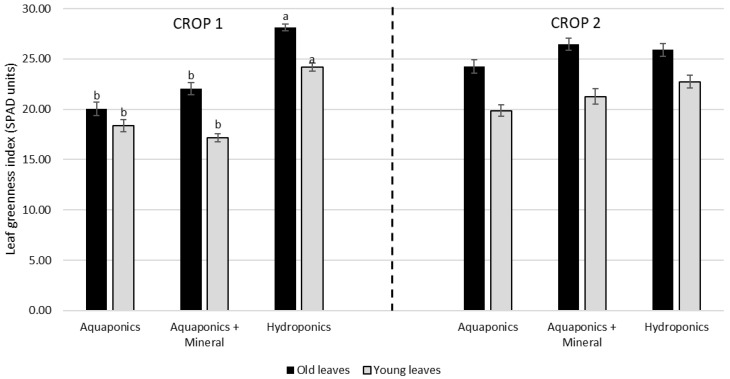
Mean (± standard error) of leaf greenness (SPAD) in young and old leaves in aquaponics, aquaponics with mineral supplementation and hydroponics (plant control). Crop one lasted 24 days and crop two lasted 27 days. Capital letters indicate differences in leaf age and lowercase letters indicate differences in production technique (two-way ANOVA, *p* < 0.05). No interaction was detected (*p* > 0.05).

Plant performance and SPAD results from crops one and two are presented in Table 3 and Figure 3. In both crops, mineral supplementation helped to improve some key phytotechnical parameters in aquaponics. In crop one (24 days), the hydroponics treatment presented higher total wet weight (256 g), total length (77 cm), root and aerial length (41 and 36 cm), length of the largest leaf (23 cm) and productivity (5 kg m^−2^), outperforming both aquaponics with and without mineral supplementation (108 and 90 g; 60 and 51 cm; 33 and 27 cm; 26 and 24 cm; 17 and 15 cm; and 2.3 and 1.9 kg m^−2^, respectively). No statistical differences were found in the number of leaves per plant (*p* > 0.05).

In crop two (27 days), an overall increase in performance was observed in both aquaponics treatments in comparison to crop one, and a decline was detected in hydroponics. However, key parameters such as total wet weight (169 g), total length (66 cm) and root length (40 cm) were higher in hydroponics, outperforming both aquaponics treatments (129 and 126 g; 63 and 55 cm; and 39 and 21 cm, respectively). Aerial length, number of leaves per plant, length of the largest leaf and productivity presented no statistical differences among treatments in crop two (*p* > 0.05).

Regarding SPAD results (Figure 3), no interaction was detected in both crops (*p* > 0.05). Crop one presented significant differences for production technique with higher SPAD values observed in hydroponics (*p* < 0.05) and with no differences between aquaponics with and without mineral supplementation (*p* > 0.05). No differences were detected in crop two (*p* > 0.05).

## 4. Discussion

### 4.1. Water Quality and Fish Performance

The water quality parameters in RAS and aquaponics (with and without mineral supplementation) remained within the recommended ranges and acceptable levels for tropical fish species [35], suggesting that these factors did not affect the fish performance. The observed values such as temperature, DO, nitrogenous compounds, alkalinity and orthophosphate were comparable to other coupled aquaponics studies using leafy greens and tropical fish species [36,37]. Although pH values were also within safety values reported for tropical species [35], this parameter likely affected the nutrient bioavailability in both aquaponics treatments, and consequently, plant growth. In coupled aquaponics the ideal pH ranges between 6.5 and 7.0, providing the best nutrient absorption by the plants, but also a functioning biofiltration to keep safe levels of un-ionised ammonia for the fish [38]. In the current study, pH > 7.0 was observed in aquaponics with and without mineral supplementation and RAS, and only hydroponics presented pH < 7.0, certainly impacting the plant performance in these treatments. At higher pH levels, many essential nutrients, particularly micronutrients, become less soluble and harder for plants to absorb. Consequently, the initial elevated pH in both aquaponics treatments likely adversely affected the availability of nutrients during both crop cycles, resulting in limited nutrient uptake and suboptimal plant growth conditions.

In terms of fish performance, both aquaponics treatments (with and without mineral supplementation) presented similar results as compared to RAS (*p* > 0.05). Survival (~100%), final individual weight (~106 g) and FCR (~1.22) were comparable among treatments, as well as with other coupled aquaponics studies using lettuce/herbs and tropical fish species [36,37]. Ani et al. [39] evaluated stocking densities of 150, 300 and 450 fish m^−3^ for 56 days in a Nile tilapia (18 g initial weight)–lettuce coupled aquaponics system. At the end of their trial, the authors found survival rates of 82–94%, final individual weights of 25–42 g and FCR of 1.4–1.9, with improved performances detected in low stocking density. Pinho et al. [36] evaluated effluents from RAS and biofloc in a coupled aquaponics system with Nile tilapia (70 g initial weight) and lettuce. At the end of the trial (21 days), the authors found survival rates above 95% in both RAS and biofloc, and final individual weight and feed conversion of 102.31 g and 1.87 and 88.12 g and 4.02 for biofloc and RAS, respectively. Although Jade perch and tilapia are different fish species, they share similar trophic levels, behaviour and physiological/nutritional aspects. Comparing our current fish performance results (63 days) with the ones using tilapia in coupled aquaponics (56 and 21 days), and considering there are no domestication or breeding efforts in Jade perch aquaculture, our results can be considered promising. However, further research is needed aiming to optimize water quality and tailor aquaponics protocols for integrated Jade perch production.

The current study also highlighted that adding lettuce to Jade perch culture did not impair the fish growth, feed efficiency and yield compared to traditional RAS. In fact, some aquaponics studies identified positive health effects when coupling fish and plant production. Baßmann et al. [40] observed that increasing the number of plants in relation to fish biomass can improve injury recovery and behavioural patterns in African catfish and basil aquaponics. The authors compared low, moderate and high densities of basil and found a higher fish welfare status in aquaponics compared with the control (RAS). Similarly, Villarroel et al. [41] observed that the addition of a commercial fertilizer sustained fish performance and greatly improved lettuce growth compared to control (with no fertilizer) and did not cause welfare implications, e.g., an increase in physiological stress levels in tilapia.

### 4.2. Nutrient Dynamics in Water, SPAD and Plant Performance

The mineral supplementation improved key macronutrient profiles in the water (e.g., K, Ca, Mg and S), resulting in enhanced plant performance in aquaponics. Although minor supplementation was performed in both crop cycles of the aquaponics + mineral treatment (only 10% of the ration utilised in hydroponics treatment), this procedure enhanced N, P, K, Mg, Ca and S concentrations in the water and positively impacted the plant yields. A similar trend was observed in the studies by Rafiee et al. [42] and Villarroel et al. [41], in which the addition of commercial fertilizers improved plant growth compared to controls (with no fertilizer) in tilapia–lettuce aquaponics. In both studies, the authors observed improvement in mineral concentrations in the water, such as N, P, K, Mg, Ca and Fe. Using molasses as a K-rich organic fertilizer, Pinho et al. [36] compared biofloc-based aquaponics versus RAS-based aquaponics. In this study, molasses helped the microbial community to thrive in biofloc and likely contributed to superior plant performance in an integrated tilapia and lettuce culture (var. red, butter and crispy) by increasing microbial mineralization of nutrients into plant-available forms.

In the current study, the hydroponics treatment outperformed the two aquaponics treatments in both crops, e.g., presenting higher total wet weight and total length. Some hypotheses suggest that a more suitable pH, fewer solids in the water (as per turbidity levels), lower sodium concentration and more balanced nutrient concentration, e.g., S and K, contributed to the enhanced performance in hydroponics.

**Figure 4:**
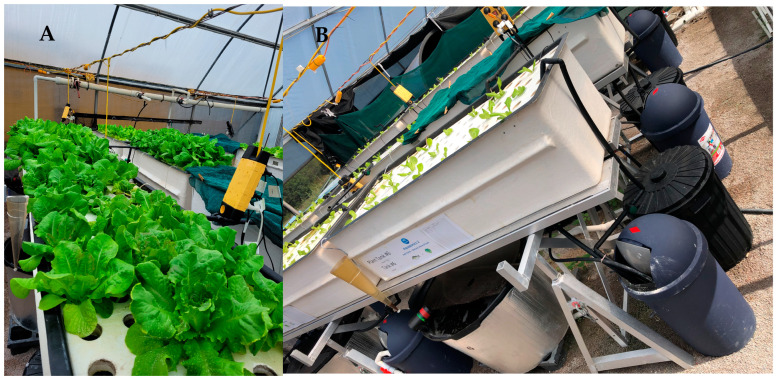
(**A**) Overall view of the visual plant quality in crop two from different treatments. (**B**) A close look at clarifiers (black compartment) and biofilters (dark blue compartment).

A deficiency of mobile nutrients, including N, K, Mg and Mo, can lead to a loss of greenness in older leaves that can be either localised or generalised. Deficiencies of immobile nutrients, including S, Cu, Fe, Mn and Zn, lead to yellowing and can impair plant growth [43]. Sulfur (S) deficiency in lettuce causes uniform chlorosis, or yellowing, across the entire leaf blade, impacting the plant growth. Additionally, potassium (K) deficiency can promote wilted plants and often cause leaf colour to turn to mottled dark green. Tips and margins can present chlorotic spots with increasing size which become necrotic and deforming over time [44]. These K and S deficiency symptoms were particularly observed in both aquaponics treatments (with and without mineral supplementation) but to a lesser extent in the second crop, suggesting a positive impact likely related to nutrient build-up, or possibly microbiome development, in the system. It is important to note that solids in RAS and both aquaponics treatments were managed and retained in the clarifiers (Figure 4). Interestingly, no visual or major accumulation was noticed, even in crop two, indicating a potential mineralization process in the clarifiers, likely helping the nutrient build-up.

It is not uncommon to find studies where hydroponics outperformed aquaponics. Ayipio et al. [45] deployed a meta-analysis to synthesise the literature on studies that compared crop yields in aquaponics and hydroponics. The overall results showed that crop yield in aquaponics was lower than in hydroponics, although no significant difference was detected. On the other hand, the opposite can also occur, especially when mineral supplementation is carried out, as observed by Delaide et al. [16]. The authors utilised high-purity mineral salts to establish a ‘complementary aquaponics solution’ using tilapia RAS effluent and observed an improvement in plant yield compared to hydroponics and aquaponics without mineral supplementation.

Nutrient supplementation is a critical consideration in aquaponics due to the inherent differences in nutrient delivery compared to hydroponics. In hydroponic systems, nutrient formulations are specifically tailored in terms of composition and concentration to provide optimal levels of macro and micronutrients for plant growth [46]. In contrast, aquaponics primarily relies on fish waste, which may not only lack certain essential nutrients but also present them in forms that are not always bioavailable to plants, potentially leading to nutrient deficiencies [47].

In the current study, we demonstrated that not only nutrient supplementation but also system maturation can play a role in improving lettuce performance in a conventional coupled aquaponics system with Jade perch. The productive performance data of lettuce collected at the end of each crop cycle, along with the fluctuation of key water parameters over time, suggest that aquaponics systems were not fully matured during crop one, despite allowing the biological filters to mature for 90 days prior to the stocking of fish and plants.

At the start-up of a new aquaponics system using the current configuration, the microbial biofilter requires several weeks to establish before the fish are introduced to ensure that the nitrifying community is active enough to efficiently convert TAN to NO_3_ to avoid ammonia and nitrite toxicity to the fish. It then normally takes several weeks after the introduction of the fish to the tanks for nutrients to build up in the system and for the complex microbiome, responsible for converting spent fish feed and waste products into soluble nutrients that can be used by the plants, to become established [23]. The time taken for the system to mature will vary depending on the fish species, stocking density, feeding rates, feed type and culture conditions. In our experiment, Figure 2B,D show that nutrient concentrations in the water column were low at the start of the experiment. When the plants were added at week 3 (Figure 2B), nitrate levels were still relatively low in all treatments, but they rapidly rose at week 4 and continued to increase in the aquaponics and RAS treatments throughout the experiment. These data suggest that insufficient nutrient accumulation prior to the first crop cycle may explain some of the difference in performance between crop one and two in the aquaponics treatments, but it is also highly likely that the establishment of the microbial community over time also boosted plant performance in the second crop cycle [23].

In crop one, the average lettuce productivity (kg m^−2^) and total wet weight (g) in the hydroponics treatment were more than twice as high, at 5.5 kg m^−2^ and 256.7 g, compared to the aquaponics without (1.9 kg m^−2^ and 91 g) and with mineral supplementation (2.3 kg m^−2^ and 108 g). In the second crop, on the other hand, the productivity of the two aquaponics treatments began to catch up with that of the hydroponics. Although their average productivity remained slightly lower, it was not statistically different from the hydroponic control. In comparison to other lettuce-based aquaponics studies, our lettuce productivity values with and without mineral supplementation (2.3–1.9 kg m^−2^) were higher than [36] and [48] in tilapia–lettuce aquaponics with 0.3 to 1.9 kg m^−2^ (21 days) and 0.3 to 0.5 kg m^−2^ (14 and 21 days), respectively. However, the season, quality and variety of lettuce seedlings, plant/fish stocking density, hydroponics technique, mineral supplementation and nutritional profile of fish feeds are factors that certainly can impact the plant performance outcomes.

Regarding the SPAD measures for both old and young leaves, mineral supplementation appeared to have no significant effect on lettuce coloration under aquaponics conditions throughout both crop cycles. However, while the greenness of plants in the aquaponics with and without mineral supplementation was significantly inferior to the ones produced in hydroponics at the end of crop one, this statistical difference was not observed in crop two. This finding suggests that system maturation likely had a more pronounced impact on improving the leaf greenness index under aquaponics conditions than mineral supplementation did.

This is also supported by the water quality parameters measured throughout both cycles, particularly electrical conductivity (EC), pH, nitrate (NO_3_) and phosphate (PO_4_). As anticipated, the EC in hydroponics remained stable throughout the 63-day experimental period. In contrast, the EC in the two aquaponics treatments and RAS increased steadily without reaching a plateau. Electrical conductivity is a key indicator of the water’s ability to conduct electricity, which is directly related to the concentration of dissolved ions in the water [46]. Higher EC values typically indicate a greater abundance of nutrients, as these dissolved ions consist primarily of essential macro and micronutrients required for plant growth. Consequently, as nutrient concentration increases, so does the conductivity of the solution. This indicates that nutrient levels accumulated in the fish-based systems as they matured, corroborated by increasing levels observed in both aquaponics and RAS treatment. However, EC values can be largely represented by sodium due to sodium-rich ingredients in fish feed formulations.

In this trial, the use of NaHCO_3_ along with CaCO_3_ to increase alkalinity in the aquaponics treatments would have contributed to the elevated sodium content in these treatments. Additionally, fish meal-based feeds (utilised in this experiment) can also contribute to increasing sodium levels in water over time. Higher sodium levels in the water can impair plant growth, as observed by [49] in a 28-day study evaluating lettuce–tilapia aquaponics in low-salinity water (3 ppt). The sodium content in the aquaponics treatment was elevated relative to the hydroponics treatment. However, it remained below the critical levels reported by Beauchamp et al. [50], who showed that significant impacts on plant performance only occurred with sodium concentrations of 400 mg L^−1^ (1200 mg L^−1^ NaCl) when EC is maintained below 4332 µS cm^−1^. In the present study, the average EC was maintained below 2500 µS cm^−1^ during crop one and trended above 3000 µS cm^−1^ during crop two in the aquaponics treatments (Figure 1). Although sodium levels accumulated over time, reaching higher levels in crop two, higher biomass was produced in both aquaponics treatments with reasonable plant visual quality (Figure 4), indicating that factors other than the sodium content and EC may have more impact on plant performance. For instance, a higher, more mature and diverse microbial community could impact the nutrient dynamics over time and, consequently, plant growth. Further studies are needed to explore the impact of alternative chemistries (e.g., CaCO_3_) for managing alkalinity levels without increasing the sodium contents of the culture water.

As per EC, the same increasing trend was also observed in NO_3_ and PO_4_ levels. Throughout the experimental period, these key indicators of effective nutrient cycling and system maturation accumulated over time, especially in RAS and aquaponics with mineral supplementation. The accumulation of NO_3_ suggests effective nitrification processes, as beneficial nitrifying bacteria convert ammonia (TAN) from fish waste into nitrites (NO_2_) and subsequently into NO_3_, which are crucial for plant growth, serving as primary nitrogen sources for healthy foliage and overall development [8]. Similarly, the rise of PO_4_, which plays a vital role in energy transfer, photosynthesis and root development, is driven by the breakdown of organic matter and the nutrient release from fish waste and uneaten feed [51].

This effect was more evident in RAS and aquaponics with mineral supplementation. As the systems matured, the increased availability of both NO_3_ and PO_4_ likely contributed to improved growth and nutrient uptake efficiency by the plants, which was evident in the enhanced lettuce performance parameters observed in aquaponics treatments during the second crop. Factors such as fish/plant species, plant/fish stocking density, mineral supplementation, biofiltration system and nutritional profile of fish feeds can have a direct impact on NO_3_ and PO_4_ dynamics in water, and, consequently, plant performance [8]. The fluctuations observed in parameters such as pH, EC, NO_3_ and PO_4_ highlight the importance of system maturation in optimising nutrient availability and promoting suitable plants within aquaponics.

## 5. Conclusions

Mineral supplementation in aquaponics helped to improve key plant production metrics while sustaining fish growth. Proper system maturation and water quality management are critical steps to ensure stable plant/fish production in aquaponics. Integrating aquaponics with Jade perch farming could provide valuable diversification opportunities and boost economic resilience for businesses in the aquaculture sector. Additionally, in light of stringent nutrient release regulations in Australia, this combination can enhance nutrient management strategies while addressing the increasing market demand for locally grown native fish.

This experiment showed that Jade perch is a robust and productive fish for aquaponics. However, it also highlighted a range of research questions that could be addressed to improve plant productivity and quality in the system. As an emerging species, Jade perch offers a unique opportunity to align ecological sustainability with economic viability, making it crucial to further explore the synergies between aquaculture and aquaponics to fully harness their potential benefits. The following are priorities focused on improving the plant yield and quality in jade perch aquaponics based on the outcomes of this trial: (i) establishing the ideal time required to allow for greater nutrient accumulation before planting the first crop, (ii) exploring the impact of increasing the feed to plant ratio on nutrient loading to the plants and fish performance, (iii) decoupling the fish and plant tanks to enable pH to be controlled at different pH set-points for the fish and plant tanks and monitoring the impact on nutrient bioavailability for the plants, (iv) lowering the sodium inputs to the system, e.g., by replacing NaHCO_3_ with other alkalinity sources, (v) investigating mineralization of solid fish waste (spent feed and faeces) to increase release of dissolved nutrients for plant uptake and (iv) exploring the role of microbiome on plant and fish health and yield.

## Figures and Tables

**Table 1:** Descriptive statistics (mean values, SD, minimum and maximum) of water quality parameters in Jade perch culture in aquaponics, aquaponics with mineral supplementation and RAS (fish control), as well as in hydroponics (plant control) during 63 days * of experimental period (crop one + crop two).

Parameter		Aquaponics	Aquaponics + Mineral	RAS	Hydroponics
Temperature AM (°C)	Mean ± SD	25.77 ± 0.94	26.11 ± 0.78	25.73 ± 0.76	26.10 ± 2.26
	*Min–max*	*23.60–28.50*	*23.80–28.40*	*23.50–28.30*	*22.11–28.20*
Temperature PM (°C)	Mean ± SD	26.25 ± 1.38	26.58 ± 1.41	26.14 ± 1.46	26.45 ± 1.43
	*Min–max*	*22.10–29.70*	*22.80–30.00*	*21.70–30.40*	*22.10–32.70*
Dissolved oxygen (mg L^–1^)	Mean ± SD	7.25 ± 0.66	7.37 ± 0.26	7.35 ± 0.69	7.87 ± 0.20
	*Min–max*	*5.82–8.06*	*6.60–8.02*	*5.66–8.05*	*7.07–8.30*
pH	Mean ± SD	7.28 ± 0.47	7.15 ± 0.48	7.65 ± 0.32	6.86 ± 0.55
	*Min–max*	*6.49–8.41*	*6.33–8.37*	*6.77–8.39*	*6.30–7.53*
Electrical conductivity (µS cm^–1^)	Mean ± SD	1922.04 ± 727.59	2234.83 ± 833.27	2229.95 ± 893.07	1218.85 ± 165.89
	*Min–max*	*348.60–3274.00*	*431.70–3693.00*	*296.00–3797.00*	*8.26–1404.00*
Turbidity (FNU)	Mean ± SD	1.77 ± 1.21	1.87 ± 1.42	1.17 ± 0.73	0.12 ± 0.23
	*Min–max*	*0.01–5.68*	*0.03–8.76*	*0.01–3.82*	*0.00–1.18*
TAN (mg L^–1^)	Mean ± SD	1.26 ± 1.29	1.63 ± 1.32	1.34 ± 1.72	2.94 ± 3.20
	*Min–max*	*0.10–4.60*	*0.04–4.00*	*0.04–8.20*	*0.08–12.00*
NO_2_ (mg L^–1^)	Mean ± SD	0.30 ± 0.24	0.28 ± 0.25	0.69 ± 0.40	0.22 ± 0.32
	*Min–max*	*0.00–0.99*	*0.00–0.93*	*0.01–1.40*	*0.00–1.15*
NO_3_ (mg L^–1^)	Mean ± SD	203.41 ± 184.45	242.67 ± 196.23	236.40 ± 185.63	182.13 ± 119.04
	*Min–max*	*0.59–650.00*	*0.74–590.00*	*2.09–620.00*	*6.00–500.00*
PO_4_ (mg L^–1^)	Mean ± SD	25.66 ± 14.43	35.57 ± 23.68	28.60 ± 18.06	51.83 ± 24.01
	*Min–max*	*0.10–56.00*	*0.15–91.00*	*0.14–69.00*	*13.00–110.00*
Alkalinity (mg CaCO_3_ L^–1^)	Mean ± SD	50.93 ± 21.53	47.92 ± 24.04	103.91 ± 54.65	50.50 ± 25.90
	*Min–max*	*15.00–95.00*	*10.00–85.00*	*10.00–215.00*	*10.00–100.00*
K (mg L^–1^)	Mean ± SD	5.26 ± 2.24	13.21 ± 2.74	9.68 ± 5.47	40.66 ± 3.55
	*Min–max*	*1.92–6.5*	*9.15–15.13*	*2.07–15.09*	*37.11–45.35*
Ca (mg L^–1^)	Mean ± SD	62.38 ± 47.49	76.08 ± 47.10	17.96 ± 1.39	45.69 ± 7.31
	*Min–max*	*16.21–127.83*	*21.93–135.44*	*16.9–19.86*	*37.61–54.88*
Mg (mg L^–1^)	Mean ± SD	10.74 ± 1.26	12.61 ± 1.67	11.58 ± 0.70	7.77 ± 0.66
	*Min–max*	*9.05–12.04*	*10.35–14.26*	*10.61–12.29*	*6.95–8.33*
S (mg L^–1^)	Mean ± SD	19.98 ± 5.06	23.51 ± 5.77	21.04 ± 5.29	33.41 ± 4.21
	*Min–max*	*13.39–25.6*	*16.10–30.01*	*14.58–27.49*	*27.71–36.94*
Na (mg/L)	Mean ± SD	152.14 ± 58.7	201.27 ± 102.4	256.65 ± 115.41	23.8 ± 12.43
	*Min–max*	*83.45–198.3*	*106.5–250.2*	*98.1–402.53*	*14.2–42.4*

* Experimental period in hydroponics: 48 days. SD: standard deviation; TAN: total ammonia nitrogen.

**Table 2:** Mean (± standard error) of productive performance of Jade perch culture in aquaponics, aquaponics with mineral supplementation and RAS (fish control) during the experimental period (63 days).

Parameter	Aquaponics	Aquaponics + Mineral	RAS	*p*-Value
Final ind. weight (g)	104.77 ± 0.90	105.45 ± 1.13	107.24 ± 1.08	0.523
SGR (%/day)	3.14 ± 0.02	3.15 ± 0.01	3.17 ± 0.01	0.653
Final biomass (kg tank^−1^)	3.25 ± 0.08	3.37 ± 0.02	3.40 ± 0.04	0.466
Productivity (kg m^−3^)	13.53 ± 0.33	14.06 ± 0.10	14.15 ± 0.16	0.460
Survival (%)	100.00 ± 0.00	100.00 ± 0.00	100.00 ± 0.00	-
FCR	1.24 ± 0.02	1.23 ± 0.01	1.21 ± 0.01	0.546

**Table 3:** Mean (± standard error) of productive performance of lettuce (*Lactuca sativa*, var. butter head) in aquaponics, aquaponics with mineral supplementation and hydroponics (plant control). Crop one lasted 24 days and crop two lasted 27 days. * Total wet weight and productivity: roots and leaves combined.

	Crop One				Crop Two			
Parameter	Aquaponics	Aquaponics + Mineral	Hydroponics	*p*-Value	Aquaponics	Aquaponics + Mineral	Hydroponics	*p*-Value
Total wet weight * (g)	90.60 ^B^ ± 5.51	108.15 ^B^ ± 5.69	255.70 ^A^ ± 10.17	<0.001	126.20 ^B^ ± 10.55	128.58 ^B^ ± 9.25	169.14 ^A^ ± 8.28	<0.01
Total length (cm)	50.58 ^C^ ± 1.41	60.03 ^B^ ± 1.94	76.97 ^A^ ± 1.88	<0.001	54.92 ^C^ ± 2.15	62.85 ^B^ ± 2.31	66.11 ^A^ ± 1.54	<0.001
Root length (cm)	26.75 ^C^ ± 1.00	33.53 ^B^ ± 1.56	40.57 ^A^ ± 1.62	<0.01	31.34 ^B^ ± 1.80	39.07 ^B^ ± 2.12	40.00 ^A^ ± 1.20	<0.01
Aerial length (cm)	23.83 ^C^ ± 0.69	26.50 ^B^ ± 0.79	36.40 ^A^ ± 0.86	0.045	23.58 ± 0.84	23.78 ± 0.62	26.11 ± 0.96	NS (0.370)
Number of leaves per plant (n)	31.67 ± 2.37	36.03 ± 2.83	38.07 ± 1.88	NS (0.453)	29.50 ± 2.11	30.37 ± 1.96	30.77 ± 1.56	NS (0.918)
Length of the largest leaf (cm)	15.84 ± 0.57	17.08 ± 0.73	23.25 ± 0.95	<0.001	19.83 ± 0.66	19.80 ± 0.68	21.02 ± 0.81	NS (0.644)
Productivity * (kg m^−2^)	1.95 ^B^ ± 0.22	2.33 ^B^ ± 0.19	5.50 ^A^ ± 0.09	<0.001	2.31 ± 0.13	2.35 ± 0.44	3.09 ± 0.17	NS (0.669)

Values in the same row with different superscripts are significantly different (*p* < 0.05). NS = no significant differences.

## Data Availability

The authors declare data availability by request.
